# The Baltimore College of Dental Surgery

**Published:** 1895-06

**Authors:** 


THE BALTIMORE COLLEGE OF DENTAL SURGERY.—The Annual Commencement of 1895 was held at Fords' Grand Opera House, Thursday afternoon, March 21st. The announcement of the graduates was made by Prof. M. W. Foster, who also conferred the degree of "Doctor of Dental Surgery upon the following members of the graduating class:

M. H. Adams, Louisiana; J. G. Armacost, Maryland.; H. G. Baxter, New York; H. J. Boyd, New York; C. E. Brown, Virginia; S. P. Cronan, Connecticut; W. G. Dalzell, Louisiana; G. R. Dashiell, Texas; A. E. DeViney, Texas; H. R. Eavey, Maryland; K. W. Egerton, Maryland; F. W. Epes, Va.; C. H. Frink, Florida; J. G. Geiger, South Carolina; Fred. Hammond, Maryland; D. S. Henry, South Carolina; M. J. Hellwig, New York; H. Hoeper, Germany; H. W. Hoffer, Texas; E. G. Laflin, Connecticut; T. H.

Lowe, Massachusetts; J. W. Lylie, Mississippi; A. M. Marcy, Ohio; C. Meikle, New York; O. Morgan, Pennsylvania; J. B. Rousseau, Alabama; L. D. Reavis, Oregon; P. C. Richardson, Texas; L. C. Rink, Pennsylvania; W. R. Roland, Pennsylvania; W. H. Simpson, Massachusetts; G. F. Showers, West Virginia; B. M. Smith, Pennsylvania; E. M. Summervine, Pennsylvania; E. H. Sting, Ohio; W. M. Sturgis, Virginia; C. H. Theberath, New Jersey; A. C. Thweatt, Virginia; F. Waesche, Maryland; J. L. Walker, Virginia; R. H. Weiscotten, New York; E. Weymouth, Maine; G. W. Williams, West Virginia; J. H. Wheeler, North Carolina; H. White, Maryland.

The address to the graduates were delivered by the Rev. William Rollins Webb, and the class address by B. M. Smith, D. D. S., of Pennsylvania.

The Prizes were awarded by Dr. Chas. A. Meeker, as follows: Diamond Medal to H. Hoeper, Gold Medal to H. W. Hoffer, Operative Dentistry to J. L. Walker, Mechanical Dentistry to G. F. Showers, Essay on Orthodontia to J. B. Rousseau.

Prizes Given: First Honor, by Faculty, Second Honors, by Faculty; Operative, by S. S. White Dental Mfg. Co.; Mechanical, by Snowden & Cowman Mfg. Co.; Bridge-Work, by S. S. White Dental Mfg. Co.; Essay Prize, by Dr. J. N. Farrar; N. Y.

The whole number of matriculates for the session was 166.

